# Supplementary material for: Polyamide Microparticles with Immobilized Enological Pectinase as Efficient Biocatalysts for Wine Clarification: The Role of the Polymer Support
Source: Molecules. 2024 Dec 30;30(1):114. doi: 10.3390/molecules30010114 (PMC11722140; doi:10.3390/molecules30010114)
Supplement: Supplementary file 1 [file molecules-30-00114-s001.zip › molecules-3354724-supplementary.pdf]

Polyamide Microparticles with Immobilized Enological Pectinase as Efficient Biocatalysts for Wine Clarification: The Role of the Polymer Support

Sandra C. Oliveira, Samuel M. Araújo, Nadya V. Dencheva * and Zlatan Z. Denchev *

IPC-Institute for Polymers and Composites, University of Minho, 4800-056 Guimarães, Portugal; b8223@dep.uminho.pt (S.C.O.); a85216@alunos.uminho.pt (S.M.A.)

* Correspondence: nadiad@dep.uminho.pt (N.V.D.); denchev@dep.uminho.pt (Z.Z.D.)

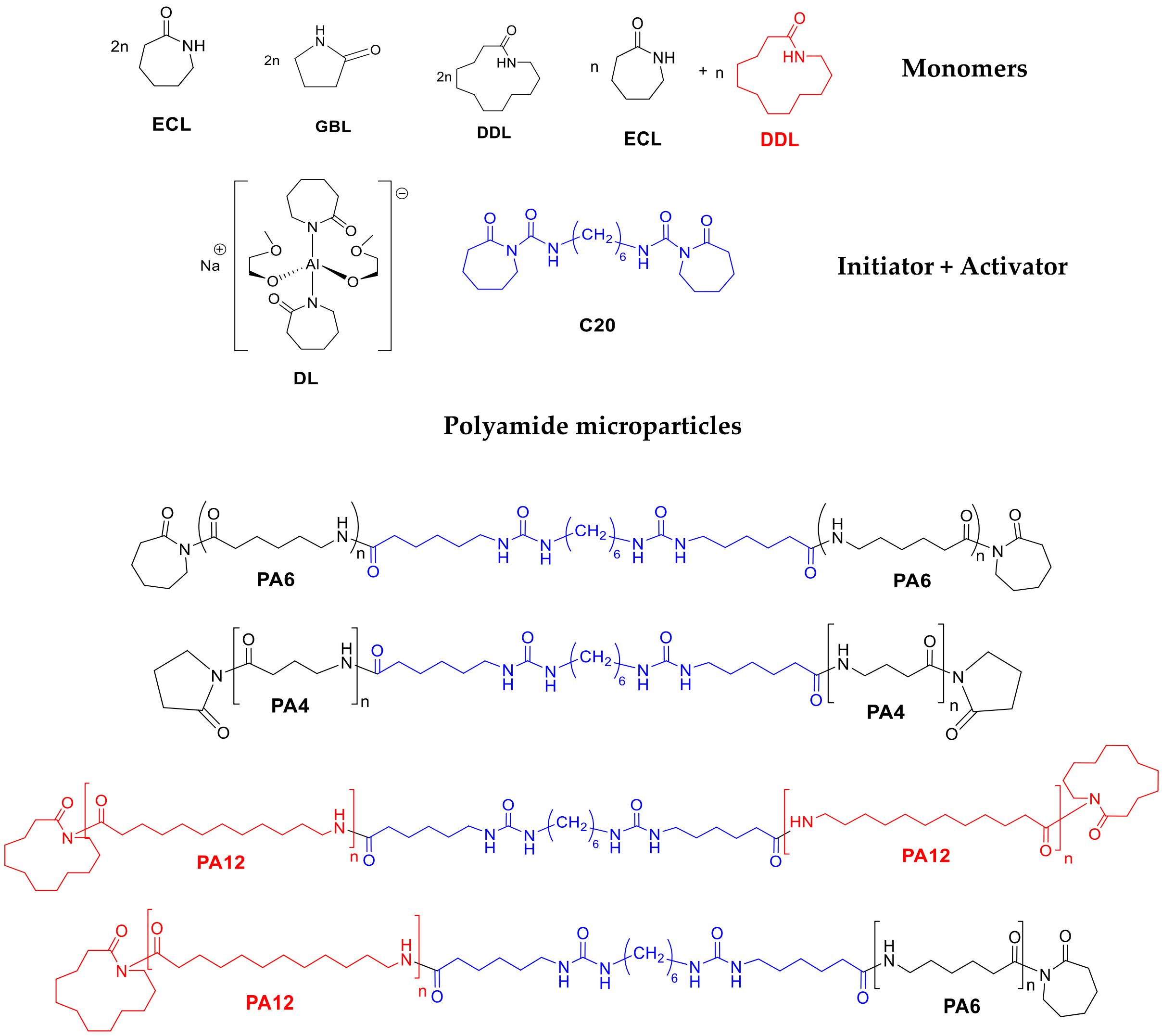

Figure S1. Chemical structures of the reagents participating in AAROP and of the microparticulate polyamides prepared. ECL = ε-caprolactam, the monomer for polyamide 6 (PA6); GBL = γ-butyrolactam, the monomer for polyamide 4 (PA4); LL = ω-laurinlactam, the monomer for polyamide 12 (PA12). C20 = the active substance of the AAROP activator; DL = the initiator of AAROP dicaprolactamato-bis-(2-methoxyethoxo)-aluminate, wherein R = $OCH_2CH2OCH_3$; PA612 = the copolymer prepared form the mixture of ECL and DDL.

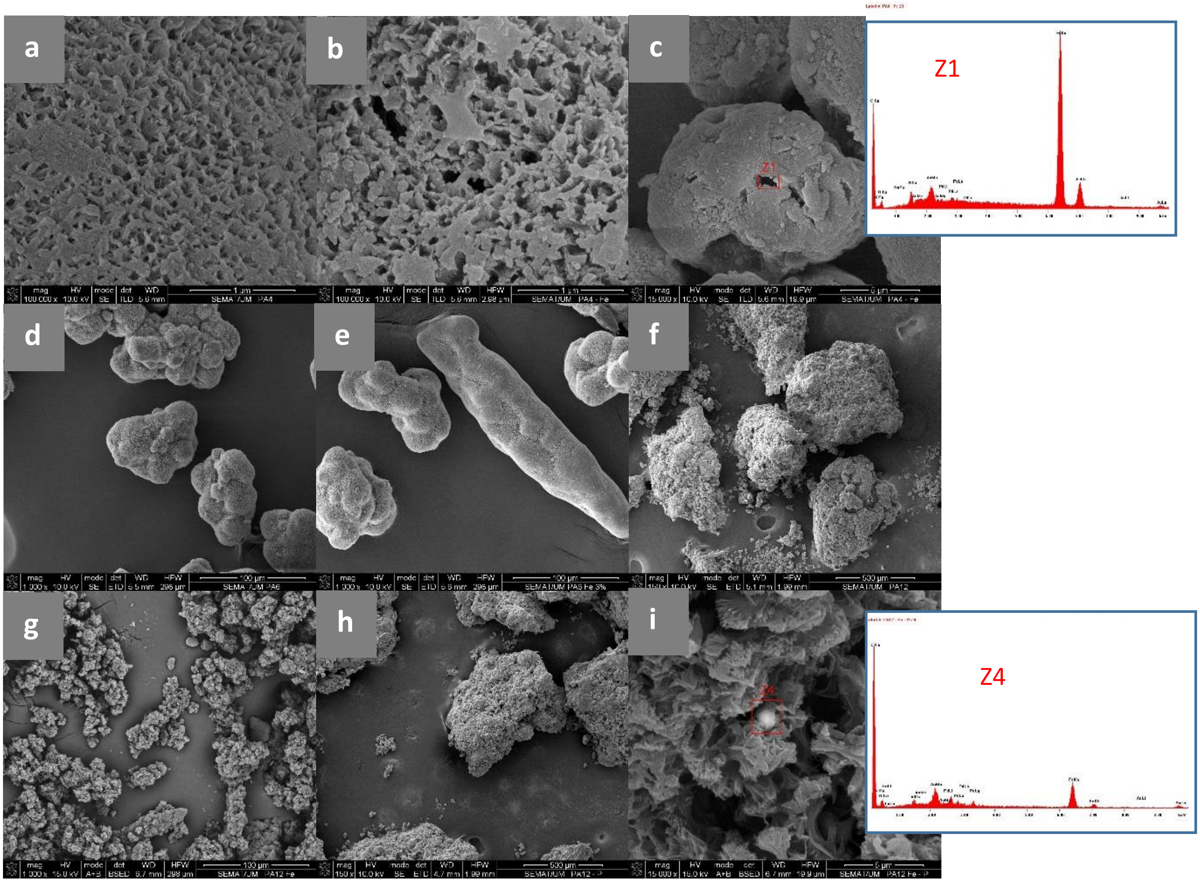

Figure S2. Selected SEM micrographs complementary to those in Figure 1 of the main text. a – PA4, close view; b – PA4-Fe, close view; c – PA4-Fe with EDX in the inset; d, e – PA6-Fe; f – PA12, low magnification; g – PA12-Fe; h – Pec@PA12; i – Pec@PA12-Fe with EDX in the inset.

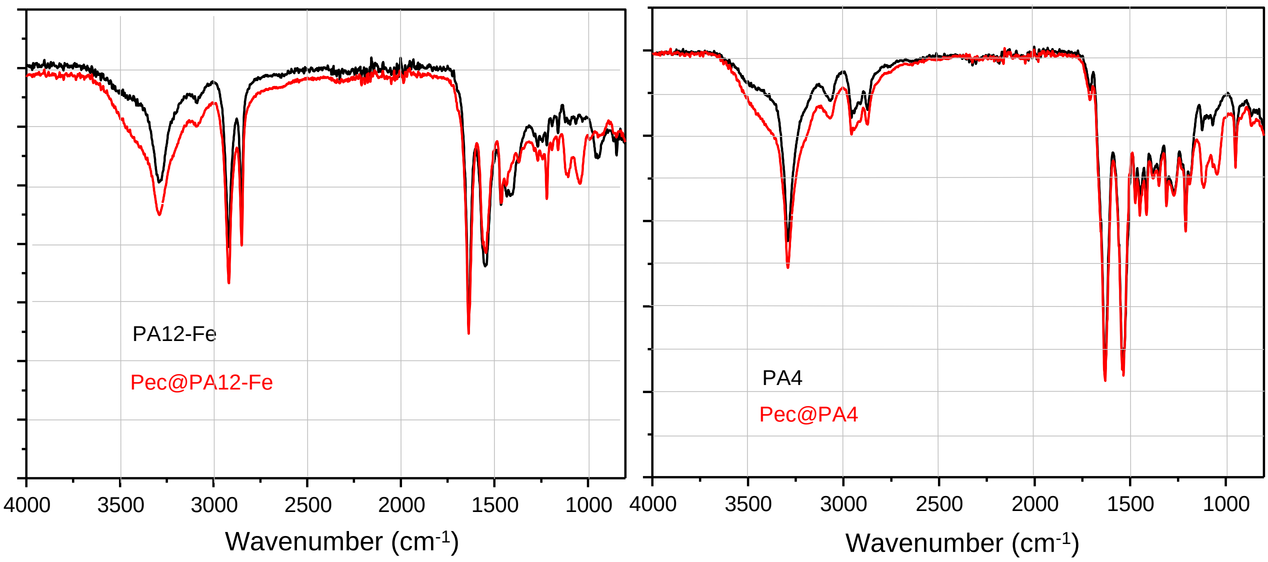

Figure S3. Selected FTIR spectra of supports PA12 and PA4 supports before and after Pec immobilization.

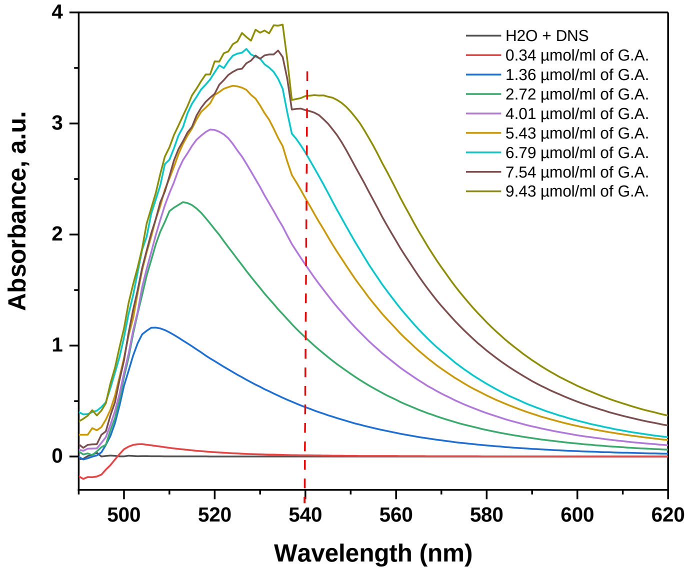

Figure S4. Evolution of the UV/VIS absorption maxima of various concentrations of galacturonic acid treated with the DNS reagent. G. A. = galacturonic acid. The vertical line at 540 nm indicates the position of the peak of the resulting 5-amino-3-nitrosalycilic acid colored product used in the construction of the standard
